# Modeling the abundance of two *Rhagoletis* fly (Diptera: Tephritidae) pests in Washington State, U.S.A.

**DOI:** 10.1371/journal.pone.0217071

**Published:** 2019-06-03

**Authors:** Tewodros T. Wakie, Wee L. Yee, Lisa G. Neven, Sunil Kumar

**Affiliations:** 1 USDA-ARS, Temperate Tree Fruit and Vegetable Research Unit, Wapato, Washington, United States of America; 2 Natural Resource Ecology Laboratory, Colorado State University, Fort Collins, Colorado, United States of America; USDA Forest Service, UNITED STATES

## Abstract

Well-adapted and abundant insect pests can negatively affect agricultural production. We modeled the abundance of two *Rhagoletis* fly (Diptera: Tephritidae) pests, apple maggot fly, *Rhagoletis pomonella* (Walsh), and western cherry fruit fly, *Rhagoletis indifferens* Curran, in Washington State (WA), U.S.A. using biologically relevant environmental variables. We tested the hypothesis that abundance of the two species is influenced by different environmental variables, based on the fact that these two species evolved in different environments, have different host plants, and that *R*. *pomonella* is an introduced pest in WA while *R*. *indifferens* is native. We collected data on fly and host plant abundance at 61 randomly selected sites across WA in 2015 and 2016. We obtained land-cover, climate, and elevation data from online sources and used these data to derive relevant landscape variables and modeled fly abundance using generalized linear models. For *R*. *pomonella*, relatively high winter mean minimum temperature, low elevation, and developed land-cover were the top variables positively related to fly abundance. In contrast, for *R*. *indifferens*, the top variables related to greater fly abundance were high Hargreaves climatic moisture and annual heat-moisture deficits (indication of drier habitats), high host plant abundance, and developed land-cover. Our results identify key environmental variables driving *Rhagoletis* fly abundance in WA and can be used for understanding adaptation of insects to non-native and native habitats and for assisting fly quarantine and management decisions.

## Introduction

The threat of invasive insects including those that threaten agriculture is a function of how well they adapt to their non-native habitats and ultimately their abundance in or near agroecosystems [1–4]. Pest species that evolved in other environments and then invade non-native regions may only develop high abundance in those localities within these regions most similar to their native habitats. These localities may differ from those occupied by native pest species that have evolved under different environmental pressures, which may be explained by adaptation to different host plant use in the case of frugivorous insects that ultimately is related to climate. From a practical standpoint, habitats where the climate and other environmental factors are conducive for favorable insect survival and thus high population build-up are areas where agricultural commodities will be most threatened. Differences among pest species in their ability to tolerate particular climates and habitats will result in different containment or management approaches for the pests.

Habitat heterogeneity and complexity as governed by vegetation type, structure, topography, and host abundance may contribute to abundance of some insect species [5]. Insects that can use a wide range of host plants or can tolerate a wide range of temperatures would be expected to have a greater distribution than those more limited in either ability. While many studies have focused on habitat heterogeneity and animal species diversity (more complex environments have more species), few have explored the relationship between habitat heterogeneity and abundance within insect species [6–8].

In the Pacific Northwest of the U.S.A., the apple maggot fly, *Rhagoletis pomonella* (Walsh) (Diptera: Tephritidae), is a major quarantine pest of commercial apple (*Malus domestica* Borkhausen), which is the top fruit commodity in the region. In Washington State (WA) alone, apple production in 2016 was valued at U.S. $2.4 billion [9]. Evidence shows *R*. *pomonella* is not native to the Pacific Northwest but was probably introduced there in infested apples from eastern North America prior to the 1970s [10,11]. In WA, *R*. *pomonella* attacks unmanaged cultivated apples, native black hawthorn (*Crataegus douglasii* Lindley) and introduced ornamental hawthorns (*Crataegus monogyna* Jacq. and *Crataegus laevigata* (Poir. DC.) [12, 13]). The abundance of *R*. *pomonella* in these host plants within WA influences their importance and threat to the apple industry, which is concentrated in the dry but irrigated central interior of the state.

Western cherry fruit fly, *Rhagoletis indifferens* Curran (Diptera: Tephritidae), is a major quarantine pest of commercial sweet cherry (*Prunus avium* (L.) L.) in the Pacific Northwest. Washington is the top sweet cherry-producing state, as sweet cherries from there were valued at U.S. $500 million in 2016 [9]. *Rhagoletis indifferens* was originally found in the foothills of the Cascade Mountain Range around the margins of the commercial cherry-growing region in central WA in its native host, bitter cherry (*Prunus emarginata* [Dougl. ex Hook.] Eaton) [14]. However, *R*. *indifferens* moved onto cultivated sweet cherry and tart cherry (*Prunus cerasus* L.) after they were introduced into the region in the mid-1800s [14,15]. The fly was first detected in a cherry-growing region in central WA in 1942 in Toppenish in Yakima County [16]. *Rhagoletis indifferens* is also found over a broad area extending to western WA [12], indicating it is capable of surviving in a wide range of climates and habitats in WA.

Previous work suggests that the most suitable habitats for *R*. *pomonella* and *R*. *indifferens* are not the same due to differences in the flies’ environmental niches [17, 18]. Within WA, there is substantial heterogeneity and diversity of habitats associated with variable climates, ranging from coastal forests and subalpine ecosystems west of the Cascade Mountain Range to ponderosa pine, bunchgrass, sagebrush, and interior cedar-hemlock ecosystems to the east [19]. Abundance patterns of *R*. *pomonella* and *R*. *indifferens* across these diverse habitats may differ. Differential tolerance to climatic factors such as temperature, precipitation, and humidity could influence the abundance of *R*. *pomonella* and *R*. *indifferens* across these habitats. *Rhagoletis pomonella* appears less able to survive dry climates than *R*. *indifferens* [20–23]. This is consistent with the hypothesis that *R*. *pomonella* survives best in climates resembling those where it evolved in eastern North America, where relative humidity is higher than in many areas of the western U.S.A. Conversely, *R*. *indifferens* evolved on bitter cherry in drier environments, such as in the aforementioned foothills of the Cascade Mountain Range, and thus may survive better in lower humidity and precipitation environments.

*Rhagoletis pomonella* is dependent mostly on *Malus* and *Crataegus* species to survive, so another logical hypothesis is that abundance of these hosts is the most important factor explaining fly abundance. However, there are instances where host tree abundance does not seem to explain fly abundance. *Rhagoletis pomonella* is rare in central WA, even where native hawthorn trees are abundant [24]. This suggests environmental factors other than host plant abundance could explain fly abundance in an area.

Host abundance should also explain the abundance of *R*. *indifferens*. However, this fly appears less abundant in western than central WA [12, 24] even though there are many unmanaged cultivated cherry and native bitter cherry trees in western WA [12]. Thus, there could be similarities and differences in the environmental factors affecting the abundance and distribution of *R*. *pomonella* and *R*. *indifferens*.

The objective of this study was to examine the role of climate and landscape heterogeneity on the abundance of *R*. *pomonella and R*. *indifferens* in WA. We tested the hypothesis that abundance of the two species is influenced by different environmental variables, specifically that moisture, cold temperature, host plant abundance and landscape heterogeneity could limit the abundance of *R*. *pomonella* while humidity, precipitation, host plant abundance, and landscape heterogeneity could limit the abundance of *R*. *indifferens*. We predict differences in fly abundance across eastern, western and central regions of WA and discuss implications of the results for adaptation of flies to non-native and native habitats and for fly quarantines and management.

## Materials and methods

**Fig 1:**
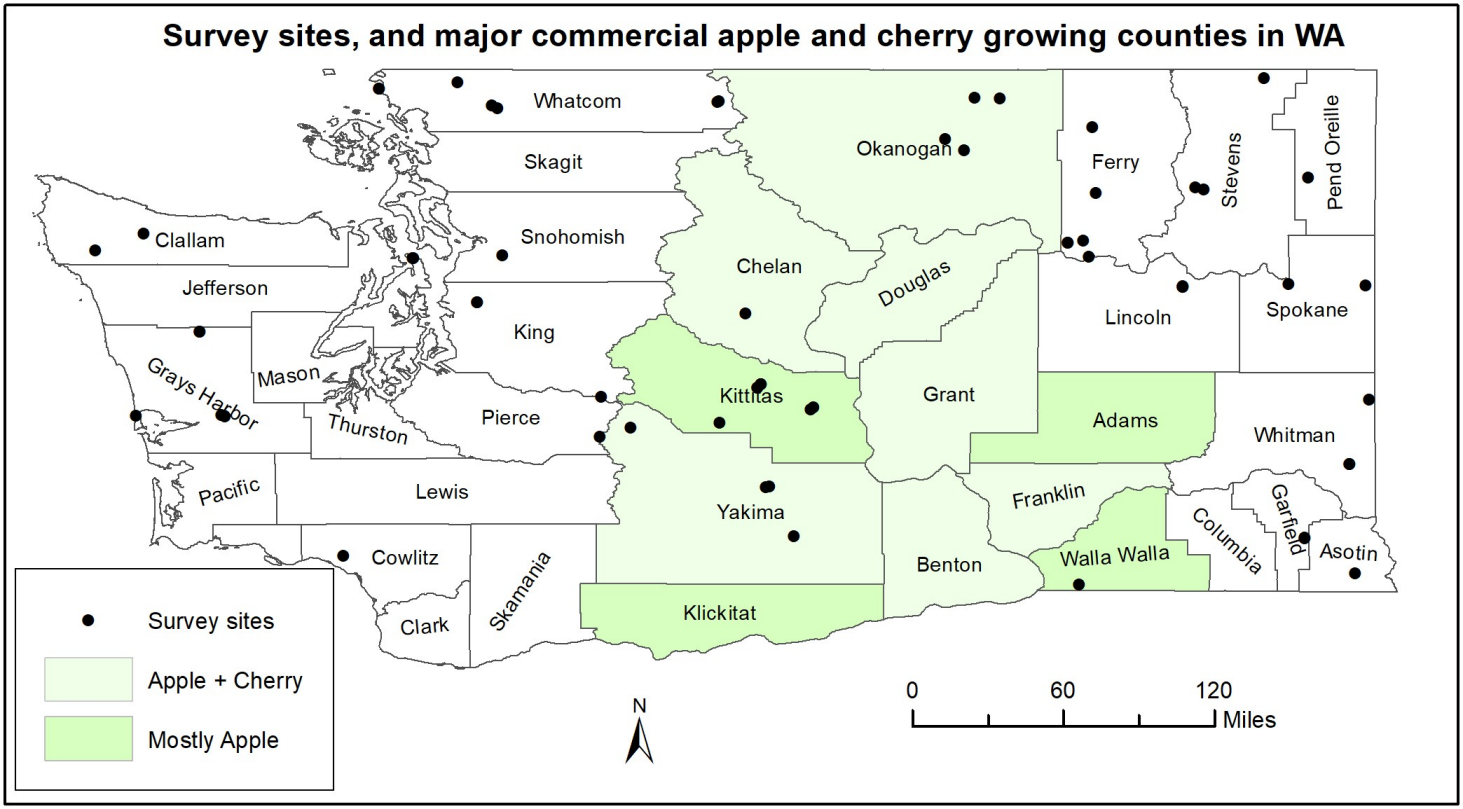
Survey sites, and major apple and cherry growing counties. Fly and host plant abundance data were collected from 61 randomly generated sites (shown in black dots) in 2015 and 2016. Major commercial apple growing regions in WA include all the major commercial cherry growing counties, and Kittitas, Adams, Klickitat and Walla Walla Counties. Major commercial cherry growing counties in WA are Yakima (28%), Chelan (18%), Benton (15%), Grant (12%), Douglas (10%), Franklin (~9%), and Okanagan (~9%). Data source: WA Apple Commission.

Fly surveys were conducted in 2015 and 2016 at 61 sites in WA using a Geographic Information System (GIS)-based stratified random sampling approach. Random samples were generated for the state of WA using the *Hawth’s analysis tools for ArcGIS* software by selecting the *generate random points* option [25]. We selected this sampling approach because the technique was found to be more efficient when compared with other statistical sampling methods [26]. Initially, more than 100 random points were generated in a GIS environment using elevation, temperature, and precipitation data layers as stratifying units (the aim was to collect environmentally heterogeneous samples). However, some of the randomly generated points were located in inaccessible sites such as a side of a cliff or a top of a mountain, thus only 61 sites in total were surveyed (Fig 1). In 2016, host plant species presence and abundance data were collected at each site by constructing 0.1-ha circular plots. Host trees used for trapping were considered the centers of the circular plots, and all host plants within the circular plot (seedlings, saplings, and mature) were counted. We represented host abundance by the total number of plants found in the circular plots, and abundance of flies by the average number of *R*. *pomonella* and *R*. *indifferens* adult flies trapped at each site throughout the study period. Flies were captured by placing a single trap per site per species.

Fly abundance data were collected from the same 61 sites using sticky yellow rectangle traps baited with ammonia odor in May to October 2015 and 2016. The Pherocon AM trap (Trecé, Adair, OK, U.S.A.) baited with the P203 AB27 lure (AgBio Inc., Westminster, CO, U.S.A.) was used for trapping *R*. *pomonella* and *R*. *indifferens*. The lure contains 27 g of ammonium bicarbonate and at 30°C has a release rate of 12.8 mg ammonium bicarbonate/h [27]. Traps were hung on host trees ≥ 1.6 m above ground and replaced every two weeks. Sites were visited 12–14 times during the trapping period. Trapping ended when the first hard freeze was recorded based on freeze indicator detectors (American Thermal Instruments, Dayton, OH, U.S.A.). Recovered traps were stored at -20°C until they could be processed. The sticky adhesive was dissolved from the traps using citrus solvent (De-Solv-It orange solution, Household products, Gilbert, AZ, U.S.A.) before flies were removed. Flies were identified to the species level and counted at the USDA-ARS Temperate Tree Fruit and Vegetable Research Unit in Wapato, WA. Pest abundance data for each species were summed and averaged over the two study years before statistical analyses were performed.

Environmental variables were obtained from different sources. The 100 m spatial-resolution elevation data set for the study site was obtained from the U.S. Geological Survey website [28]. Using this elevation data set, ArcGIS software [29], and the surface gradient and geomorphometric modeling toolbox [30], six topographic variables that represent the spatial heterogeneity of each study site were calculated. These metrics included compound topographic index, site exposure index, surface roughness, flow direction, slope, and aspect. Furthermore, we used the U.S. National Land-Cover Database to represent the different land-cover classes found at the study sites [31].

The dispersal distances of both *R*. *pomonella* and *R*. *indifferens* adult flies are relatively short. Previous studies indicate most *R*. *pomonella* flies are found within ~60 m of release locations [32], while most *R*. *indifferens* flies are recovered within ~36 m of release locations [33]. Given the relatively short dispersal distances of the two flies, we represented landscape heterogeneity using land-cover proportions close to the trap sites. Proportions were calculated in an area having a circular radius of 120 m (2 X 60 m), and with the trap trees at the center. The randomly generated 61 trap sites fell on seven land-cover categories, including developed (open space, low-intensity, medium-intensity, high-intensity), cultivated crop, evergreen forest, grassland-herbaceous, herbaceous-wetland, pasture-hay, and shrub-scrub categories (S1 Appendix). However, when we considered the 120 m-radius circular area, additional land-cover categories were identified. These included open water, barren-land, woody wetlands, mixed forest, and deciduous forest categories (S1 Appendix). We aggregated closely related land-cover categories before statistical analyses. All developed land-cover categories were reclassified as developed, all forest classes were grouped into forest, and all wetland classes were reclassified as wetland.

The climatic conditions of the study sites were represented using climate variables obtained from the ClimateWNA program [34, 35]. ClimateWNA uses the Parameter-Elevation Relationships on Independent Slopes Model (PRISM) baseline information [36] to generate high-resolution climate data for western North America. The program can be used to generate scale-free climate data for any location by specifying latitude, longitude, and elevation. Here, normal, annual, and seasonal variables for the most current time period, 1981–2010, were used. Using geographically referenced trap site locations and the elevation data set, more than 100 biologically relevant climate variables were extracted from the ClimateWNA program. These included 23 annual and 60 seasonal climate variables (S2 Appendix). The ClimateWNA data were effectively used to predict the geographic distribution of invasive cheatgrass (*Bromus tectorum* L.) in Colorado, U.S.A. [37], and the mountain pine beetle (*Dendroctonus ponderosae* [Hopkins]) in western North America [38].

Relationships with host availability, climate variability, and landscape heterogeneity have been modeled using different approaches including linear models and their extensions [3, 39]. In this study, *R*. *pomonella* and *R*. *indifferens* abundance at the 61 locations were modeled using host abundance data, landscape metrics, and topographic and climate variables. We used linear models for preliminary analysis and investigated the magnitude and direction of relationships among the response and predictor variables (each variable was tested separately). Abundance data were log-transformed (log_10_+1) prior to linear modeling. This preliminary analysis helped us identify the important variables and reduced the variables from more than one-hundred to just 20. In addition, cross-correlation tests conducted in R statistical software [40] indicated most of the ClimateWNA variables to be highly correlated (Tables 1 and 2). Here we explained the observed abundance of *R*. *pomonella* and *R*. *indifferens*, choosing one representative variable out of each set of highly correlated variables (Pearson’s correlation *r* ≥ ± 0.7) from the models. Despite a slightly higher correlation with the climate variables, we kept elevation in one of the models (the *R*. *pomonella* model), because elevation improved model results when either used separately or in combination with other variables.

**Table 1:** Pearson’s correlations (r) and p values for *R*. *pomonella*. Results show that the climate variables were correlated with each other. Tmin_wt = winter mean minimum temperature, DD5_wt = winter degree-days below 5°C, FFP = frost free period, Host = host plant abundance, Elev. = elevation, D = developed, S.S. = shrub-scrub, G.H. = grassland-herbaceous, P.H. = pasture-hay, C.C. = cultivated crops, Forest = evergreen, deciduous, and mixed forests, and W = wetland. Barren land and open water land-cover categories, which were both non-correlated and not included in the analysis, are not shown here due to space limitations. Complete descriptions of climate and land-cover variables are presented in S1 and S2 Appendices.

		EMT	Tmin_wt	DD5_wt	Host	FFP	Elev.	D	Forest	S.S.	G.H.	P.H.	C.C.	W
EMT	r													
	p													
Tmin_wt	r	1.00												
	p	0.00												
DD5_wt	r	0.96	0.94											
	p	0.00	0.00											
Host	r	-14.00	-0.13	-0.19										
	p	0.28	0.32	0.05										
FFP	r	0.94	0.94	0.91	-0.26									
	p	0.00	0.00	0.00	0.05									
Elev.	r	-0.84	-0.84	-0.80	0.18	-0.89								
	p	0.00	0.00	0.00	0.16	0.00								
D	r	0.36	0.36	0.34	0.00	0.36	-0.35							
	p	0.00	0.00	0.01	0.99	0.00	0.01							
Forest	r	-0.21	-0.21	-0.18	-0.01	-0.34	0.35	-0.36						
	p	0.11	0.11	0.06	0.90	0.01	0.05	0.00						
S.S.	r	-0.39	-0.39	-0.42	-0.07	-0.29	0.25	-0.45	-0.23					
	p	0.00	0.00	0.00	0.58	0.02	0.05	0.00	0.08					
G.H.	r	-0.26	-0.28	-0.19	-0.08	-0.17	0.24	-0.17	-0.19	0.19				
	p	0.04	0.03	0.14	0.51	0.19	0.06	0.18	0.15	0.15				
P.H.	r	0.43	0.42	0.49	-0.07	0.39	-0.37	-0.06	-0.16	-0.18	-0.14			
	p	0.00	0.00	0.00	0.59	0.00	0.00	0.64	0.21	0.16	0.28			
C.C.	r	-0.06	-0.04	-0.13	0.04	-0.01	-0.05	-0.10	-0.25	-0.18	-0.13	-0.10		
	p	0.65	0.73	0.33	0.77	0.94	0.69	0.42	0.04	0.17	0.31	0.45		
W	r	0.21	0.20	0.24	0.29	0.13	-0.19	-0.06	-0.10	-0.11	-0.16	0.19	-0.14	
	p	0.11	0.12	0.07	0.02	0.31	0.13	0.67	0.13	0.40	0.20	0.13	0.28	

**Table 2:** Pearson’s correlation (r) and p values for *R*. *indifferens*. Results show that most of the climate variables were correlated with each other. SHM = summer heat-moisture index, AHM = annual heat-moisture index, CMD = Hargreaves climatic moisture deficit, CMD_wt = winter Hargreaves climatic moisture deficit, CMD_sm = summer Hargreaves climatic moisture deficit, Eref = Hargreaves reference evaporation, Host = host plant abundance, Forest = evergreen, deciduous and mixed forests, D = developed, S.S. = shrub-scrub, G.H. = grassland herbaceous, P.H. = pasture-hay, C.C. = cultivated crops, and W = wetland. Complete descriptions of climate and land-cover variables are listed in S1 and S2 Appendices.

		SHM	AHM	CMD	CMD_wt	Eref	CMD_sm	Hosts	D	Forest	S.S.	G.H.	P.H.	C.C.	W
SHM	r														
	p														
AHM	r	0.94													
	p	0.00													
CMD	r	0.91	0.93												
	p	0.00	0.00												
CMD_wt	r	0.73	0.65	0.48											
	p	0.00	0.00	0.00											
Eref	r	0.74	0.78	0.77	0.47										
	p	0.00	0.00	0.00	0.00										
CMD_sm	r	0.87	0.89	0.99	0.41	0.75									
	p	0.00	0.00	0.00	0.00	0.00									
Host	r	0.07	0.04	0.08	-0.07	-0.08	0.09								
	p	0.59	0.75	0.52	0.59	0.53	0.49								
D	r	0.12	0.02	-0.03	0.33	0.14	-0.05	-0.09							
	p	0.37	0.86	0.80	0.01	0.28	0.68	0.50							
Forest	r	-0.31	-0.32	-0.29	-0.18	-0.42	-0.26	0.22	-0.36						
	p	0.10	0.01	0.02	0.17	0.00	0.04	0.90	0.00						
S.S.	r	0.21	0.29	0.37	-0.17	0.19	0.37	0.10	-0.45	-0.23					
	p	0.10	0.02	0.00	0.09	0.14	0.00	0.45	0.00	0.08					
G.H.	r	-0.10	0.01	0.03	-0.11	-0.09	0.01	0.00	-0.17	-0.19	0.19				
	p	0.45	0.94	0.83	0.38	0.50	0.95	0.98	0.19	0.15	0.15				
P.H.	r	-0.25	-0.27	-0.33	-0.07	-0.07	-0.33	-0.08	-0.06	-0.16	-0.18	-0.14			
	p	0.04	0.03	0.01	0.58	0.58	0.01	0.54	0.64	0.21	0.15	0.28			
C.C.	r	0.40	0.38	0.37	0.25	0.40	0.37	-0.04	-0.10	-0.25	-0.18	-0.13	-0.10		
	p	0.00	0.00	0.00	0.50	0.00	0.00	0.77	0.42	0.04	0.17	0.31	0.45		
W	r	-0.20	-0.20	-0.25	-0.09	-0.09	-0.26	0.07	-0.60	-0.10	-0.11	-0.16	0.19	-0.14	
	p	0.12	0.11	0.05	0.48	0.51	0.03	0.57	0.66	0.43	0.41	0.20	0.13	0.29	

For the final analyses, generalized linear models (GLM) were selected based on the assumption that our non-transformed fly count data have a Poisson distribution. GLM fits less-complex models, and its results are relatively easy to interpret. The statistical analyses were conducted in R statistical software environment using the *glm* function and R packages including *stats*, *MASS*, and *Hmisc* [40]. We ran several GLM models using different combinations of variables and selected the best model using lowest Akaike’s Information Criterion (AIC) values. AIC, which penalizes complex models, allows the selection of the most parsimonious model among a set of different models [41, 42].

To test for differences in *R*. *pomonella* and *R*. *indifferens* abundance across western, central, and eastern WA, the mean numbers of flies caught on traps per county in each region were calculated. The numbers of counties included in the study within each region were replicates. Western WA comprised seven counties, central WA four counties, and eastern WA eight counties (western: King, Whatcom, Cowlitz, Snohomish, Clallam, Gray’s Harbor, and Kitsap; central: Kittitas, Yakima, Chelan, and Okanogan; eastern: Lincoln, Walla Walla, Garfield, Asotin, Stevens, Ferry, Spokane, and Pend Oreille). Within each county, there were one to nine sites (= traps). Means for each county were calculated based on data from the two seasons. Because the data did not meet the assumption of normality, a Kruskal-Wallis test was conducted on rankings of abundance within each species across regions.

## Results

### *Rhagoletis* *pomonella*

The most important variables for explaining *R*. *pomonella* abundance in the final GLM model included winter mean minimum temperature (Tmin_wt), elevation, and four different land-cover types (Table 3). Host plant abundance was an important variable that was positively associated with *R*. *pomonella* abundance, but it was not as important as winter temperature. Furthermore, the AIC of the GLM model increased when host plant was included in the model. All land-cover categories included in the GLM model were positively associated with *R*. *pomonella* abundance. The developed, cultivated crops, and pasture-hay categories were highly significant while the shrub-scrub category was moderately significant (Table 3).

**Table 3:** GLM model results for *R*. *pomonella*. Significant variables include winter mean minimum temperature (Tmin_wt), land-cover classes (developed, shrub-scrub, cultivated crops, pasture-hay), and elevation. Though extreme minimum temperature over 30 years (EMT), winter degree days below 5°C (DD5_wt), and frost-free period (FFP) were important, we did not include these variables in the model due to cross-correlations.

Coefficients	Estimate	Std. Error	Z Value	Pr (>|z|)
Intercept	-1.89	0.69	-2.71	0.0067 **
Tmin_wt	0.257	0.076	3.397	0.0006 ***
Elevation	-0.002	0.0008	-2.14	0.032 *
Developed	4.77	0.696	6.858	< 0.0001 ***
Shrub-scrub	3.158	1.33	2.37	0.017*
Cultivated crops	5.26	0.798	6.594	< 0.0001 ***
Pasture-hay	2.95	0.78	3.769	0.0001 ***

Significance codes:

*** = 0.001,

** = 0.01,

* = 0.05.

**Fig 2:**
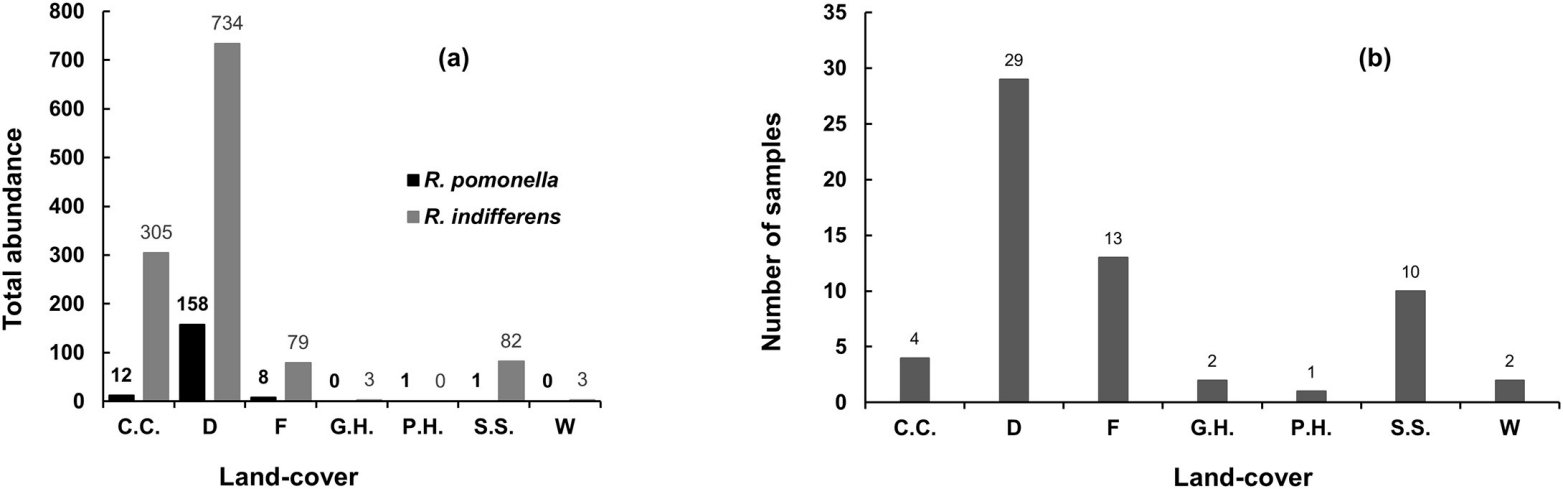
Abundance of *R*. *pomonella* and *R*. *indifferens* in Washington State, U.S.A. (A) = total abundance by land-cover, and (B) = sample frequency by land-cover. C.C = cultivated crops; D = developed areas; F = forest; G.H. = grassland-herbaceous; P.H. = pasture-hay; W = wetland; S.S. = shrub-scrub.

In the linear models, elevation was negatively associated with fly abundance, as were low mean minimum temperatures, low winter degree-days below 5°C, and low numbers of frost-free days (S1 Fig). Other than elevation, the topography or landscape metrics considered in this study did not have a significant role in explaining the distribution of *R*. *pomonella*. Based on percent land-cover (Fig 2a and 2b), *R*. *pomonella* has relatively high abundance in developed areas (consistent with Table 3); relatively low abundance in the cultivated crops and forests; very low abundance in pasture-hay and shrub-scrub categories; and zero or near zero abundance in the other land-cover categories.

**Fig 3:**
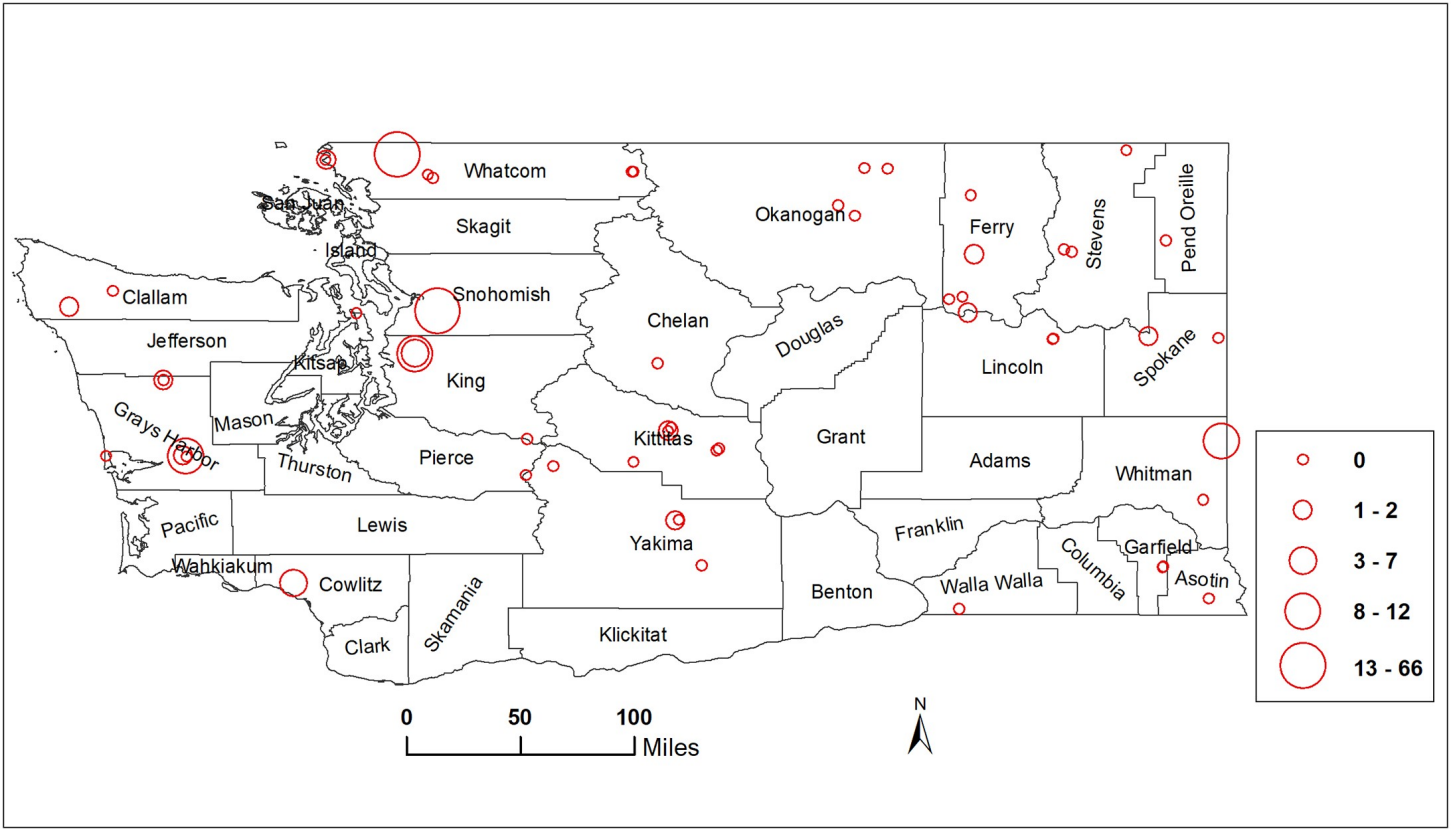
Abundance of *R*. *pomonella* in Washington State, U.S.A. Abundance data collected at 61 sites in 2015 and 2016 were averaged and classified into 5 classes using the natural breaks classifier in ArcGIS [43].

Our results indicate that western WA counties had greater *R*. *pomonella* abundance than central and eastern WA counties (Fig 3). Statistical tests indicated that the number of *R*. *pomonella* caught on traps in western WA was significantly greater than in central and eastern WA, based on mean ranks of counts (14.5, 7.5, and 7.3, respectively: *χ*^2^ = 7.66; df = 2; *P* = 0.0217). The mean numbers of *R*. *pomonella* caught ± SE in western, central, and eastern WA were 12.6 ± 9.0, 0.1 ± 0.1, and 0.6 ± 0.5, respectively. There was no difference in numbers of flies caught in central and eastern WA.

### *Rhagoletis* *indifferens*

The most important variables for explaining *R*. *indifferens* abundance in the GLM model included winter Hargreaves climatic moisture deficit (CMD_wt), annual heat-moisture index (AHM), host plant abundance, and three different land-cover types (Table 4). Among the land-cover categories, developed areas, shrub-scrub, and cultivated crops were positively associated with *R*. *indifferens* abundance and were highly significant. The grassland-herbaceous and wetland categories were both significant but negatively associated with *R*. *indifferens* abundance (Table 4).

**Table 4:** GLM model results for *R*. *indifferens*. Significant variables include winter Hargreaves climatic moisture deficit (CMD_wt), annual heat-moisture index (AHM), land-cover (developed, shrub-scrub, cultivated crops, grassland-herbaceous, wetlands) and host abundance. Though Hargreaves climatic moisture deficit (CMD), summer heat-moisture index (SHM), and Hargreaves reference evaporation (Eref) were also significant, we did not include these variables in the model due to cross-correlations.

Coefficients	Estimate	Std. Error	Z Value	Pr(>|z|)
Intercept	0.91	0.17	5.38	< 0.0001 ***
CMD_Wt	0.86	0.04	20.91	< 0.0001 ***
AHM	-0.06	0.004	-16.62	< 0.0001 ***
Host abundance	0.04	0.002	19.14	< 0.0001 ***
Developed	3.94	0.212	18.6	< 0.0001 ***
Shrub-scrub	4.86	0.28	17.17	< 0.0001 ***
Cultivated Crops	6.13	0.25	24.29	< 0.0001 ***
Grassland-herbaceous	-1.65	0.53	—3.10	< 0.0018 **
Wetlands	-5.45	1.01	-5.41	< 0.0001 ***

Significance codes:

*** = 0.001,

** = 0.01,

* = 0.05.

In the linear models, unlike for *R*. *pomonella*, as the moisture deficit increased (areas become drier), the abundance of *R*. *indifferens* also increased (S2 Fig). Specifically, annual and summer Hargreaves climatic moisture deficit (CMD_sm and CMD), summer heat-moisture index (SHM_sm), and Hargreaves reference evaporation (Eref), were positively associated with *R*. *indifferens* abundance (S2 Fig). Also unlike for *R*. *pomonella*, elevation did not play a significant role in explaining the distribution of *R*. *indifferens* in this study. Based on percent land-cover (Fig 2), the abundance of *R*. *indifferens* was highest in developed areas; medium in cultivated crops and forest; low in the wetlands and shrub-scrub areas; and zero in the other land-cover categories.

**Fig 4:**
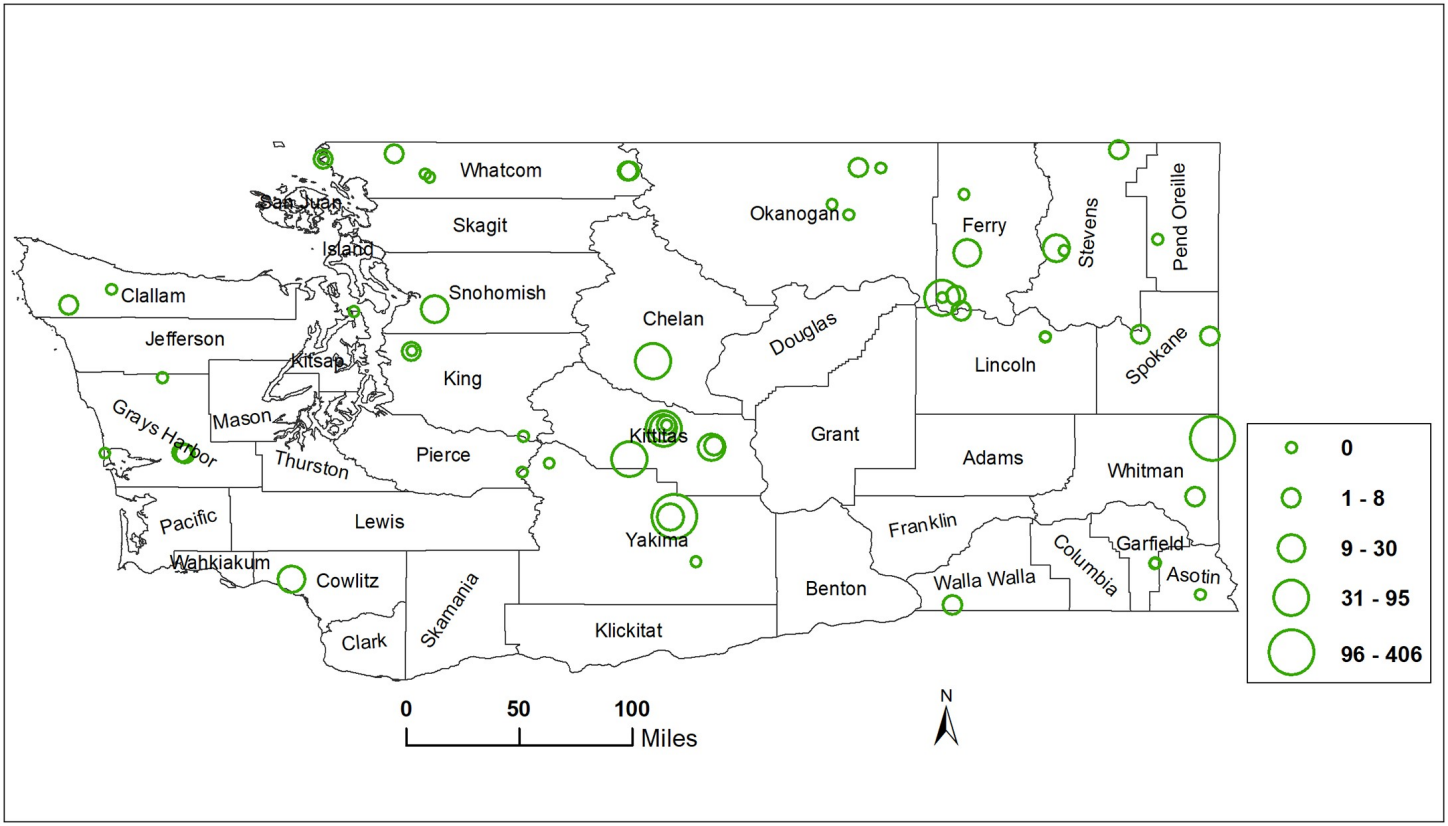
Abundance of *R*. *indifferens* in Washington State, U.S.A. Abundance data collected from 61 sites in 2015 and 2016 were averaged and classified into 5 classes using the natural breaks classifier in ArcGIS [43].

In contrast to *R*. *pomonella* (Fig 3), *R*. *indifferens* abundance was greater in central than western WA (Fig 4). The number of *R*. *indifferens* caught in central WA was numerically greater than in western and eastern WA, although differences were not significant based on mean ranks of counts (14.4, 9.1, and 8.6, respectively; *χ*^2^ = 3.13; df = 2; *P* = 0.2088). The mean numbers of *R*. *indifferens* caught ± SE in central, western, and eastern WA were 50.4 ± 21.6, 7.1 ± 3.9, and 17.5 ± 12.6, respectively.

## Discussion

The results of our study support the hypothesis that abundance of *R*. *pomonella* and *R*. *indifferens* is influenced by different environmental variables. Results show how two members within a genus of frugivorous insects can have different habitat requirements to maximize population growth, as measured by abundance, even though they are found in broadly overlapping habitats. This may not be surprising given the relatively distant phylogenetic positions of the two species within *Rhagoletis* [44] and their evolution on host plants with different fruiting phenologies, which probably influences their ability to survive in different habitats. Ultimately as shown in this study, *R*. *pomonella* is more abundant in the relatively wet region of western WA than drier central WA while the reverse is true of *R*. *indifferens*.

### Drivers of *R*. *pomonella* abundance

Our analyses show that *R*. *pomonella* in WA is most abundant and therefore survives optimally in areas with warmer winters. These areas are the lower-lying regions west of the Cascade Mountain Range. The lower abundance and apparent poorer survival of *R*. *pomonella* in colder regions may be related to where the fly evolved in eastern North America. Winter temperatures where *R*. *pomonella* evolved in eastern North America are generally warmer than those at higher elevations in WA [45–48]. However, the presence of flies in colder sites such as Ellensburg in the current study suggests there may be subpopulations of flies in eastern North America (the presumed original source of WA flies) that are adapted to low temperatures.

Elevation was the most important landscape metric related to *R*. *pomonella* abundance, probably because higher elevation is directly related to lower temperatures. Ultimately, *R*. *pomonella* may have poorer survival in the colder, drier upland forests or foothills of the Cascade Mountains than in the warmer, lower elevations west of the Cascades. Fly abundance in relation to three measures of temperature (S1 Fig) are all consistent with cold adversely affecting *R*. *pomonella* survival. In addition, areas that are cold but where there is no snow cover in some years due to a drier environment can lead to lower soil temperatures in the winter [49], further reducing *R*. *pomonella* survival.

Host plant abundance was an important variable explaining *R*. *pomonella* abundance in the GLM and linear models, but it was not among the top variables in either model. Thus, results suggest that high abundance of hawthorn, apple, or both hosts in an area will not necessarily result in a high abundance of flies if the climate in that area is too cold. This implies that climate or habitat suitability differences can exist between an invasive insect such as *R*. *pomonella* and its hosts native to the area. This is important for predicting the potential abundance of a non-native insect even when suitable, or marginally suitable, hosts are present. This interpretation is consistent with data showing low or no *R*. *pomonella* infestations in hawthorn or apple fruit in WA and Montana even where hawthorn tree abundance is high [10,12,50,51], although host fruit suitability (not examined here) could also be a factor.

Surprisingly, the moisture variables (e.g., mean annual precipitation and mean annual summer precipitation; winter, spring, summer, and autumn relative humidity) were not among the top predictors of *R*. *pomonella* abundance, suggesting the fly can tolerate environments drier than predicted by laboratory experiments [20, 21]. However, proportion of developed area, which include housing units and therefore human settlement and irrigation, was one of the important metrics in the GLM model. This could mean that human settlement, and therefore managed, irrigated habitats, could increase *R*. *pomonella* abundance by allowing host trees to survive where otherwise they would not.

In addition to the developed category, shrub-scrub, cultivated crops, and pasture-hay categories were also positively significant, even though fly abundance in the latter was low (Fig 2). For shrub-scrub and pasture-hay categories, presence of an individual fly affected the analysis, probably because the frequencies of sites sampled in these categories were low. One link across shrub-scrub, cultivated crops, and pasture-hay categories may be that they are associated with natural moisture or irrigation that allow host hawthorn or apple trees to survive. However, the fact that shrub-scrub was the least significant of the three (*P* = 0.017) suggests that lack of human intervention by way of irrigation or planting of host trees slightly reduces the chances of large *R*. *pomonella* populations establishing.

### Drivers of *R*. *indifferens* abundance

In contrast to *R*. *pomonella*, for *R*. *indifferens* most of the climatic variables had a positive relationship with abundance. The top variables most positively related to *R*. *indifferens* abundance in WA in the linear model were related to evapotranspiration and moisture deficit, i.e., drier conditions. These results suggest dry conditions are more conducive to high *R*. *indifferens* abundance than wetter conditions. Evapotranspiration is the sum of evaporation and plant transpiration from the Earth’s land and ocean surface to the atmosphere, called the reference evapotranspiration (ET_0_) [52–54]. Although these moisture deficit variables are not based on soil moisture (or lack thereof) that would have the largest impact on fly survival, as 10–11 months of the fly’s life cycle are spent as a puparium in soil [14], air vapor deficits are positively correlated with low soil moisture, particularly in arid and semi-arid regions [55]. Consistent with results here, soils do not need to be irrigated for survival of central WA-origin *R*. *indifferens* [23]. Furthermore, inspection of the distribution of *R*. *indifferens* abundance across WA (Fig 4) indicates that fly abundance is relatively low in western WA where it is wet and high in central WA where it is dry, visually supporting the statistical analyses.

Cherry host abundance was one of the significant variables in the GLM model, suggesting areas that are hot and dry with the most unmanaged cherry trees will have the greatest *R*. *indifferens* abundance. However, host abundance was not among the top variables related to *R*. *indifferens* abundance in the linear model, suggesting that there are differences in fly abundance given equal tree abundance between western and central WA. Cherry trees in western WA seem abundant enough to support larger fly populations, yet fly populations there are lower than in central WA. Drier climate positively affecting host fruit loads and other ecological differences may be reasons. Under the relatively wet, humid, and warm winter climate in western WA, cherry set may be lower than in central WA; trees may be subject to more fungal growth, e.g., brown rot (*Monilinia* spp.) [56,57], and thus less healthy than trees in central WA. Finally, birds may remove large proportions of the relatively few cherries in some western WA trees, depriving flies of developmental sites (W. L. Y., personal observations).

The developed land-cover category was significant in the GLM model, as this variable, also discussed in relation to *R*. *pomonella* above, is associated with residences, lawn grasses, and thus irrigated habitat. Furthermore, as for *R*. *pomonella*, the cultivated crops category was significant as well, probably because cultivated sweet cherry trees are common in this land-cover class. This suggests the abundance of *R*. *indifferens* is dependent on the availability of sweet cherries rather than irrigation per se. For example, if urban areas were planted with less preferred host plants than sweet cherry, then perhaps the developed land-cover class would not be significant due to lower host quality. If true, then the significance of the developed land-cover class is more likely due to host plant quality than irrigation. The categories grassland-herbaceous and wetlands were highly significant but negatively so, likely because most host trees are unable to survive in these habitats.

Results indicate that planting of cultivated cherry trees in dry, hot irrigated habitat had unanticipated consequences for pest management. The ability of *R*. *indifferens* to survive in drier habitats with high sweet cherry abundance has increased the range and abundance of the fly since pre-human settlement. There may be more *R*. *indifferens* now in WA than before colonists from the eastern U.S. arrived with their cultivated cherry trees and began irrigating the region in the mid-1800s [15].

### Implications for fly quarantines and management

Our results can be used in fly management efforts. The goal for managing *R*. *pomonella* is to keep it out of commercial apple orchards to maintain export markets [50, 58]. There has never been a report of a *R*. *pomonella* larva in commercially-packed apples from WA [59]. Implications for management of *R*. *pomonella* for apple export purposes include risk assessments for apple orchards and establishment of Areas of Low Pest Prevalence or Pest Free Areas (ALPP or PFAs) [60], which would allow movement of apples without cold treatment. Apple plantings in areas with especially cold winters may have lower risk of being infested simply because of low or no *R*. *pomonella* in those areas. In our study, apparently due in large part to low winter temperatures, no *R*. *pomonella* were detected in Okanogan, Stevens, Pend Oreille, Asotin, Garfield, Chelan and Walla Walla Counties. Washington State Department of Agriculture did detect *R*. *pomonella* in Okanogan County in 2017 [61] after our study, but our results suggest the fly is unlikely to establish large populations there, so the county could in the future be designated an ALPP for apple export purposes. Our study also suggests removal of unmanaged host trees around orchards in moderately warm sites may limit fly abundance.

*Rhagoletis indifferens* is occasionally found infesting commercial cherries at low levels [62], due mostly to its high abundance in unmanaged trees near some cherry orchards. Unlike *R*. *pomonella*, ALPP and PFAs do not seem possible for *R*. *indifferens* in the dry commercial cherry-growing areas in central WA as it is abundant there. To reduce the threat of *R*. *indifferens* invading and then infesting cherries in orchards, unmanaged cherry trees within 0.29 km of cherry orchards (maximum dispersal distance of *R*. *indifferens* determined by Jones and Wallace [63]) need to be removed. Cherry orchards themselves need to be properly managed using approved insecticides applied on schedule; cherries remaining on trees postharvest need to be sprayed with insecticides or removed [64]. Our findings do not alter these practices, but they do show that they will almost certainly need to continue, and that any new cherry plantings in drier areas in eastern WA where there are no flies now could be threatened in the future.

## Summary and conclusions

In summary, we used primary survey data, biologically relevant environmental variables, and modeling approaches to assess the abundance of two *Rhagoletis* pests in WA. Furthermore, we explained the observed abundance and distribution patterns of these pests in relation to climatic factors, host abundance, land-cover, and landscape metrics. Our results identify pest abundant sites and can be used for understanding adaptation of insects to non-native and native habitats and for aiding fly quarantine and management decisions. Experimental work on proximate and ultimate causes of the relationships we established in this study will be needed to more fully understand the intricacies of factors affecting fly population abundance.

## Supporting information

S1 Fig Relationships among environmental variables and *R*. *pomonella* abundance in Washington State, U.S.A. (A) elevation had a negative relationship with *R*. *pomonella* abundance while (B) winter mean minimum temperature (Tmin_wt), (C) winter degree days below 5°C (DD5_wt), and (D) frost-free period (FFP) had positive relationships with *R*. *pomonella* abundance.
(TIF)

S2 Fig Relationship among environmental variables and *R*. *indifferens* abundance in Washington State, U.S.A. Most of the climatic variables used in the analysis had a positive relationship with *R*. *indifferens* abundance, suggesting that moisture deficit plays a significant role in explaining the distribution of the pest in the state. (A) CMD = Hargreaves climatic moisture deficit, (B) CMD_wt = winter Hargreaves climatic moisture deficit, (C) SHM = summer heat-moisture index, (D) Eref = Hargreaves reference evaporation.
(TIF)

S1 Appendix Land-cover classes and descriptions. The following land-cover categories were found within 125 m radius of the trap locations.
(DOCX)

S2 Appendix ClimateWNA variables and descriptions. The following annual and seasonal climate variables were obtained using ClimateWNA program—V.5.30.
(DOCX)
