# Supplementary figures and images for: Modeling the abundance of two Rhagoletis fly (Diptera: Tephritidae) pests in Washington State, U.S.A
Source: PLoS One. 2019 Jun 3;14(6):e0217071. doi: 10.1371/journal.pone.0217071 (PMC6546340; doi:10.1371/journal.pone.0217071)

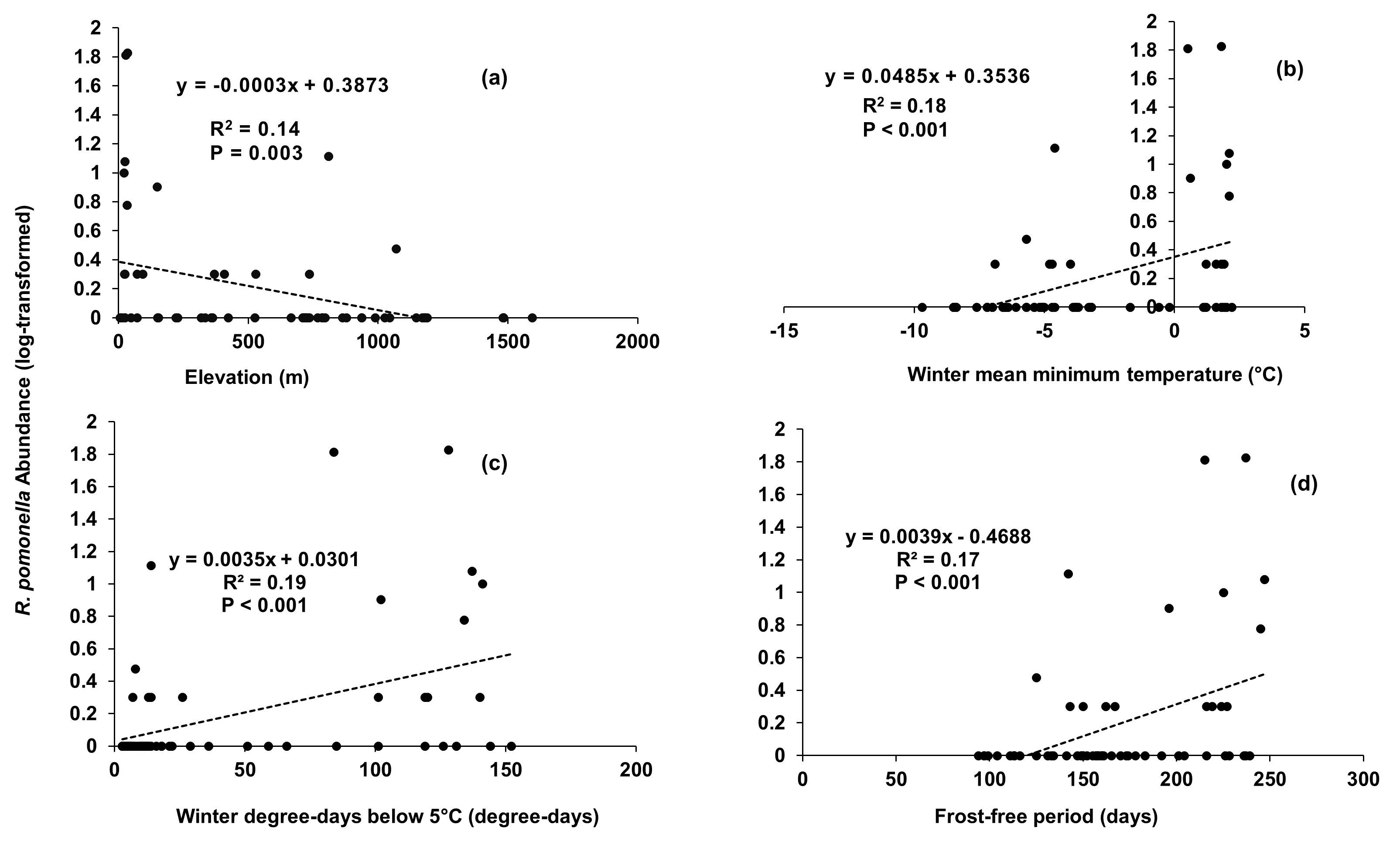

Supplement: S1 Fig — (A) elevation had a negative relationship with R. pomonella abundance while (B) winter mean minimum temperature (Tmin_wt), (C) winter degree days below 5°C (DD5_wt), and (D) frost-free period (FFP) had positive relationships with R. pomonella abundance. (TIF) [file pone.0217071.s001.tif]

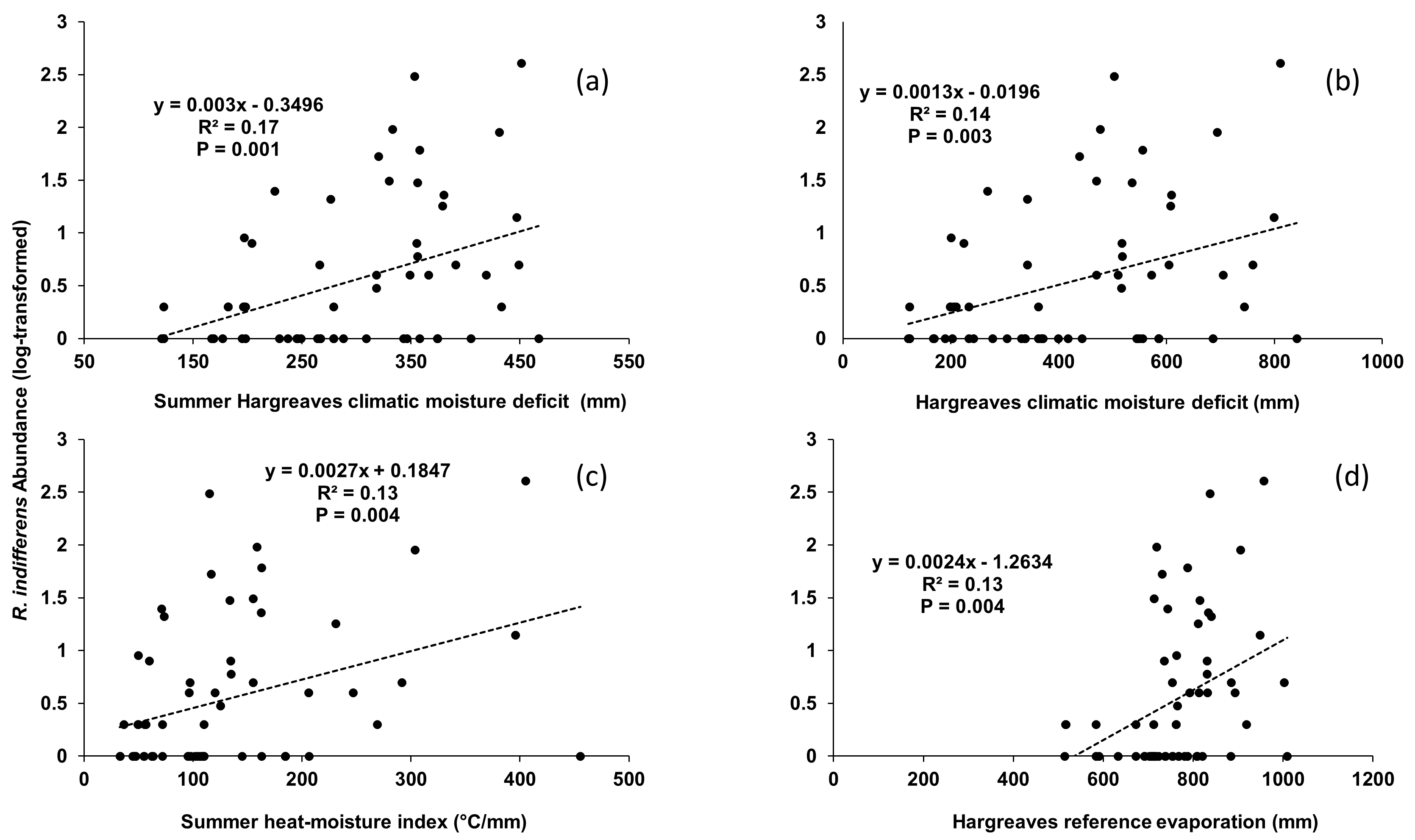

Supplement: S2 Fig — Most of the climatic variables used in the analysis had a positive relationship with R. indifferens abundance, suggesting that moisture deficit plays a significant role in explaining the distribution of the pest in the state. (A) CMD = Hargreaves climatic moisture deficit, (B) CMD_wt = winter Hargreaves climatic moisture deficit, (C) SHM = summer heat-moisture index, (D) Eref = Hargreaves reference evaporation. (TIF) [file pone.0217071.s002.tif]
