# Supplementary material for: Modeling the abundance of two Rhagoletis fly (Diptera: Tephritidae) pests in Washington State, U.S.A
Source: PLoS One. 2019 Jun 3;14(6):e0217071. doi: 10.1371/journal.pone.0217071 (PMC6546340; doi:10.1371/journal.pone.0217071)
Supplement: S1 Appendix — The following land-cover categories were found within 125 m radius of the trap locations. (DOCX) [file pone.0217071.s003.docx]

| Class | Classification Description |
| --- | --- |
| Developed, Open Space | Areas with a mixture of some constructed materials, but mostly vegetation in the form of lawn grasses. Impervious surfaces account for less than 20% of total cover. These areas most commonly include large-lot single-family housing units, parks, golf courses, and vegetation planted in developed settings for recreation, erosion control, or aesthetic purposes. |
| Developed, Low Intensity | Areas with a mixture of constructed materials and vegetation. Impervious surfaces account for 20% to 49% percent of total cover. These areas most commonly include single-family housing units. |
| Developed, Medium Intensity | Areas with a mixture of constructed materials and vegetation. Impervious surfaces account for 50% to 79% of the total cover. These areas most commonly include single-family housing units. |
| Developed, High Intensity | Highly developed areas where people reside or work in high numbers. Examples include apartment complexes, row houses and commercial/industrial. Impervious surfaces account for 80% to 100% of the total cover. |
| Deciduous Forest | Deciduous Forest - Areas dominated by trees generally greater than 5 meters tall, and greater than 20% of total vegetation cover. More than 75 percent of the tree species shed foliage simultaneously in response to seasonal change. |
| Evergreen Forest | Areas dominated by trees generally greater than 5 meters tall, and greater than 20% of total vegetation cover. More than 75% of the tree species maintain their leaves all year. Canopy is never without green foliage. |
| Mixed Forest | Mixed Forest - Areas dominated by trees generally greater than 5 meters tall, and greater than 20% of total vegetation cover. Neither deciduous nor evergreen species are greater than 75 percent of total tree cover. |
| Shrub/Scrub | Areas dominated by shrubs; less than 5 meters tall with shrub canopy typically greater than 20% of total vegetation. This class includes true shrubs, young trees in an early successional stage or trees stunted from environmental conditions. |
| Grassland/Herbaceous | Areas dominated by graminoid or herbaceous vegetation, generally greater than 80% of total vegetation. These areas are not subject to intensive management such as tilling but can be utilized for grazing. |
| Pasture/Hay | Areas of grasses, legumes, or grass-legume mixtures planted for livestock grazing or the production of seed or hay crops, typically on a perennial cycle. Pasture/hay vegetation accounts for greater than 20% of total vegetation. |
| Cultivated Crops | Areas used for the production of annual crops, such as corn, soybeans, vegetables, tobacco, and cotton, and also perennial woody crops such as orchards and vineyards. Crop vegetation accounts for greater than 20% of total vegetation. This class also includes all land being actively tilled. |
| Emergent Herbaceous Wetlands | Areas where perennial herbaceous vegetation accounts for greater than 80% of vegetative cover and the soil or substrate is periodically saturated with or covered with water. |
| Woody Wetlands | Woody Wetlands - Areas where forest or shrub land vegetation accounts for greater than 20 percent of vegetative cover and the soil or substrate is periodically saturated with or covered with water. |
| Open Water | All areas of open water, generally with less than 25% cover or vegetation or soil. |
| Barren Land | Barren Land (Rock/Sand/Clay) - Barren areas of bedrock, desert pavement, scarps, talus, slides, volcanic material, glacial debris, sand dunes, strip mines, gravel pits and other accumulations of earthen material. Generally, vegetation accounts for less than 15% of total cover. |
