# Supplementary material for: Modeling the abundance of two Rhagoletis fly (Diptera: Tephritidae) pests in Washington State, U.S.A
Source: PLoS One. 2019 Jun 3;14(6):e0217071. doi: 10.1371/journal.pone.0217071 (PMC6546340; doi:10.1371/journal.pone.0217071)
Supplement: S2 Appendix — The following annual and seasonal climate variables were obtained using ClimateWNA program—V.5.30. (DOCX) [file pone.0217071.s004.docx]

**1) Annual variables**

*Directly calculated annual variables*:

MAT = mean annual temperature (°C),

MWMT = mean warmest month temperature (°C),

MCMT = mean coldest month temperature (°C),

TD = temperature difference between MWMT and MCMT, or continentality (°C),

MAP = mean annual precipitation (mm),

MSP = mean annual summer (May to Sept.) precipitation (mm),

AHM** = annual heat-moisture index (MAT+10)/(MAP/1000))

SHM** = summer heat-moisture index ((MWMT)/(MSP/1000))

*Derived annual variables:*

FFP** = frost-free period (measured in days = eFFP-bFFP)

bFFP = the day of the year on which FFP begins (estimated using polynomial functions)

eFFP = the day of the year on which FFP ends (estimated using polynomial functions)

EMT** = extreme minimum temperature over 30 years. EMT was calculated in ClimateWNA using the following equation:

EMT = -18.09995 + T_min(1) X_ 2.14095 + T^2^_(min1)_ x 0.06836 + T^2^ _min(12)_ X (-0.04771) +TD^2^ x 0.00306. Where, T_min(1)_ and T_min(12)_ are the average minimum temperatures (°C) for January and December, respectively. TD is the temperature difference (°C) between mean warmest and coldest monthly temperature.

Eref** = Hargreaves reference evaporation (mm)

Eref(m) = E_Har(m)_ x (1.18 – 0.0065 x latitude). Where the latitude is in degrees. Eref(m) is summed over the months of the year to give Efef output by ClimateWNA.

E_Har(m)_ = the efference monthly evaporation from the Hargreaves equation (in mm) was calculated in ClimateWNA using the following equation:

for T_m_ ≥ 0, E_Har(m)_ = 0.0023 x d x S_0_ x [T_m_ +17.8] X T^0.5^_r(m);_ for T_m_ < 0, E_Har(m)_ = 0

where d is the number of days in the month; S_0_ is the water equivalent of the radiation above the atmosphere (mm/day) at the latitude of the site for the day of the year in the middle of the month; T_m_ is the monthly mean daily temperature (°C); and T_r(m)_ is the mean daily temperature range (°C), that is, the difference between the monthly mean maximum and minimum temperatures (Hargreaves and Samni 1982, Wang 2012, Wang et al. 2016).

CMD** = Hargreaves climatic moisture deficit (mm) was calculated in ClimateWNA using the following equation:

for Eref ≤ *P*_(m)_, CMD_(m)_ = 0; for Eref_(m)_ > *P*_(m),_ CMD_(m)_ = Efef_(m)_ - *P*_(m)_ where *P*_(m)_ stands for monthly precipitation and Eref_(m)_ stands for monthly reference evaporation. The annual climatic moisture deficit is the sum of the monthly moisture deficits.

RH = mean annual relative humidity (%)

**2) Seasonal variables**

*Seasons:*

Winter (_wt): Jan, Feb, Dec

Spring (_sp): March, April and May

Summer (_sm): June, July and August

Autumn (_at): September, October and November

*Directly calculated seasonal variables:*

Tmin_wt** = winter mean minimum temperature (°C)

*Derived seasonal variables:*

DD5_wt** = winter degree-days below 5°C (degree day). This was calculated in ClimateWNA using monthly average temperature values, the number of days in a month, and a polynomial function.

CMD_wt** = winter Hargreaves climatic moisture deficit (mm)

CMD_sp = spring Hargreaves climatic moisture deficit (mm)

CMD_sm** = summer Hargreaves climatic moisture deficit (mm)

CMD_at = autumn Hargreaves climatic moisture deficit (mm)

** = indicates important variables used in the analysis.
